# Supplementary material for: Unveiling the Lewis Acid Catalyzed Diels–Alder Reactions Through the Molecular Electron Density Theory
Source: Molecules. 2020 May 29;25(11):2535. doi: 10.3390/molecules25112535 (PMC7321097; doi:10.3390/molecules25112535)
Supplement: Supplementary file 1 [file molecules-25-02535-s001.pdf]

Supplementary Materials

Unveiling the Lewis Acid Catalyzed Diels–Alder Reactions Through the Molecular Electron Density Theory

Luis R. Domingo [1,*] Mar Ríos-Gutiérrez [1,2] and Patricia Pérez [3]

[1] Department of Organic Chemistry, University of Valencia, 46100 Burjassot, Valencia, Spain; rios@utopia.uv.es

[2] Department of Chemistry and Chemical Biology, McMaster University Hamilton, Ontario L8S 4L8, Canada

[3] Department of Chemistry, Computational and Theoretical Chemistry Group, Faculty of Sciences, University Andres Bello, 8370146 Santiago, Chile; p.perez@unab.cl

* Correspondence: domingo@utopia.uv.es

Index

S2 1. Classifications of the Diels–Alder reactions.

S6 2. Study of the P-DA reaction between Cp **1** and acrolein **7**.

S7 3. BET study of the P-DA reaction between Cp **1** and complex **7-BF$_3$**.

S10 4. References

S11 Figure with the B3LYP/6-311G(d,p) gas phase geometries of the regio- and stereoisomeric TSs and involved in the P-DA reaction of Cp **12** with complex **7-BF$_3$**.

S12 Figure with the B3LYP/6-311G(d,p) gas phase geometries of the regio- and stereoisomeric TSs and intermediates involved in the P-DA reaction of Cp **14** with complex **7-BF$_3$**.

S13 Table with the B3LYP/6-311G(d,p) gas phase total energies of the stationary points involved in the P-DA reactions between Cp **1** and acrolein **7**, and the series of LA complexes.

S13 Table with the B3LYP/6-311G(d,p) gas phase total energies of the stationary points involved in the P-DA reactions of Cps **12** – **14** with complex **7-BF$_3$**.

S14 Table with the B3LYP/6-311G(d,p) total energies, enthalpies, entropies, and Gibbs-free energies, computed at 25 °C in DCM, of the stationary points involved in the P-DA reaction of Cp **13** with complex **7-BF$_3$**.

1. Classifications of the Diels–Alder Reactions

The Diels–Alder (DA) reaction is one of the most studied organic reactions from a synthetic as well as a theoretical viewpoint. DA reactions have several types of classifications depending on diverse experimental and theoretical criteria. Herein, some of them are recompiled as they are used in the manuscript.

1.1. Classification of the DA Reactions Within the MEDT

According to the polar character of DA reactions, they have been classified as [1]:

- Non-polar DA (N-DA) reactions, such as the DA reactions between butadiene or cyclopentadiene with ethylene;
- Polar DA (P-DA) reactions, such the DA reactions of cyclopentadiene with a series of electrophilic cyanoethylene derivatives; and
- Ionic DA (I-DA) reactions, such as the DA reactions between cyclopentadiene as iminium cations (charged electrophiles).

This is a useful classification because the feasibility of a DA reaction depends on the polar character of the reaction [1]. Note that N-DA reactions, such as that between butadiene and ethylene, do not take place easily in an experimental setting. I-DA reactions are the extreme of P-DA reactions in which one of the two reagents is an ionic species.

In turn, depending on the direction of the flux of the electron density, DA reactions can be classified into [2]:

- P-DA reactions of forward electron density flux (FEDF) are those when the electron density fluxes from the diene, acting as a nucleophile, to the ethylene, acting as an electrophile;
- P-DA reactions of reverse electron density flux (REDF) are those when the electron density fluxes from the ethylene, acting as a nucleophile, to the diene, acting as an electrophile; and
- N-DA reactions are classified as of null electron density flux (NEDF).

This classification, which is arbitrary, makes it possible to easily characterize some P-DA reactions such as that between cyclopentadiene and nitroethylene yielding the [4 + 2] cycloadduct as the FEDF, in which the diene is the nucleophile, or that yielding the [2 + 4] cycloadduct as the REDF, in which the heterodiene is the electrophile (see Scheme S1).

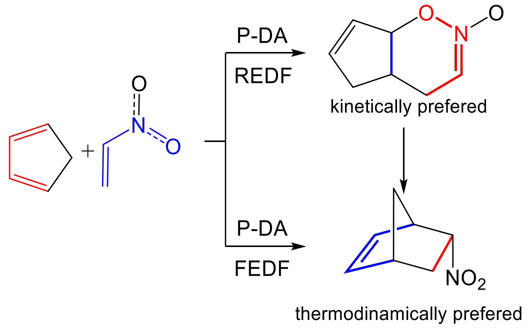

Scheme S1. Polar Diels–Alder (P-DA) reactions of reverse electron density flux (REDF) and of forward electron density flux (FEDF).

Note that this classification is easily established since cyclopentadiene always acts as a nucleophile and nitroethylene as an electrophile.

The analysis of the global electron density transfer [3] (GEDT) at the transition state structures (TSs) characterizes the non-polar or polar character of a DA reaction, as well as the direction of the electron density flux. On the other hand, the analysis of the global conceptual DFT [4] (CDFT) indices, such as the electronic chemical potential μ, the electrophilicity ω index, and the nucleophilicity *N*

index at the ground state of the reagents, also makes it possible to classify the DA reactions into these groups.

1.2. Classification of the DA Reactions by the Molecular Mechanism

Depending on the number of elementary steps in which the DA reaction takes place, it can be classified as:

- One-step reaction when the formation of two single bonds takes place in an elementary step; and
- Two-step reaction when the formation of two single bonds takes place in two different elementary steps, with the formation of a diradical or zwitterionic intermediates.

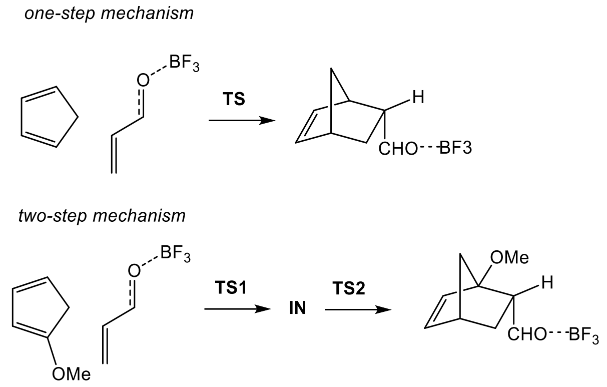

Scheme S2. One-step and two-step mechanisms involved in DA reactions.

In turn, the one-step reactions can be classified depending on the synchronicity in the formation of the two single bonds into:

- *Synchronous one-step reaction*, such as the DA reactions of cyclopentadiene with ethylene or tetracyanoethylene, in which the two single bonds are simultaneously formed;
- *Asynchronous one-step reaction*, such as the DA reactions of cyclopentadiene with acrolein or cyanoethylene, in which the formation of a single bond is more advanced with respect to the other single bond; and
- *Two-stage one-step reaction*, such as the DA reactions of cyclopentadiene with of 1,1-dicycanoethylene, in which the formation of the second single bond begins when formation of the first single bond is already completed [5].

1.3. Classification of the Competitive Reaction Paths of DA Reactions

The DA reaction between the non-symmetric diene **I** and ethylene **II** given in Chart S1 can yield until $2^5 = 32$ different cycloadducts through 32 competitive reaction paths.

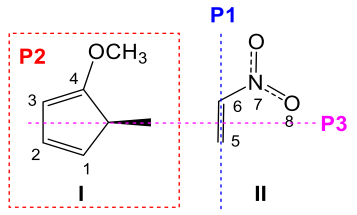

Chart S1. Symmetry planes of the reagents involved in P-DA reactions.

The different reaction paths are classified as:

- Pairs of *endo* and *exo* stereoisomeric reaction paths resulting in the approach of the C5-C6 double bond of ethylene **II** to the diene **I**, through the two faces of ethylene **II**. The *endo* approach mode corresponds to the approach of the $–NO_2$ group of ethylene **II** above the two double bonds of diene **I**. The presence of stereoisomeric reaction paths demands that the ethylene **II** be non-symmetric through plain **P1**;

- Pairs of diastereoisomeric reaction paths resulting in the approach of the C5-C6 double bond of ethylene **II** through the two faces of diene **I**. These reaction paths are only possible when one of the two reagents is chiral. The presence of diastereoisomeric reaction paths demands that the diene **I** be non-symmetric through plain **P2**;

- Pairs of regioisomeric reaction paths resulting in interactions between the C1 and C5, and the C4 and C6 centers, or the C1 and C6, and the C4 and C5 centers. The presence of regioisomeric reaction paths demands that both reagents are non-symmetric through plain **P3**;

- Pairs of chemoisomeric reaction path resulting in the participation of the C5–C6 or N7–O8 double bonds of nitroethylene. The presence of chemoisomeric reaction paths demands that the ethylene have more than one double bond; and

- Pairs of *pseudocyclic* reaction paths yielding [4 + 2] or [2 + 4] cycloadducts [6]. The presence of *pseudocyclic* reaction paths demands that the ethylene have more than one conjugated double bond. Note that formation of the [4 + 2] or [2 + 4] cycloadducts in the P-DA reaction given in Scheme S1 can be considered as an example of *pseudocyclic* selectivity.

The most common cases are DA reactions of symmetric dienes with monosubstituted ethylenes such as the reaction of Cp **1** with acrolein **7**, which presents only a pair of *endo*/*exo* stereoisomeric reaction paths (see Scheme S4), or DA reactions of non-symmetrically 1- or 2-substituted dienes with monosubstituted ethylenes, which have four competitive reaction paths: two pair of *endo*/*exo* stereoisomers, and two pairs of regioisomers (see Scheme S3).

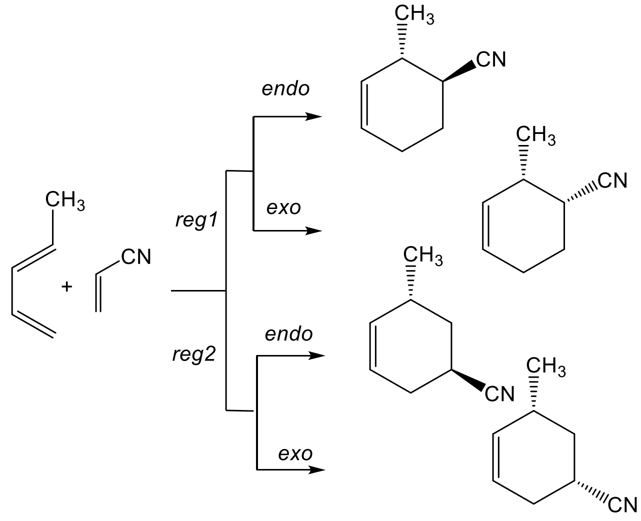

Scheme S3. Stereo- and regioisomeric reaction paths involved in the DA reaction of 1,3-pentadiene with acrylonitrile.

Depending on the relative position of the substituents in the two reagents, the regioisomeric reaction paths are named as *ortho*, *meta* and *para* (see Chart S2).

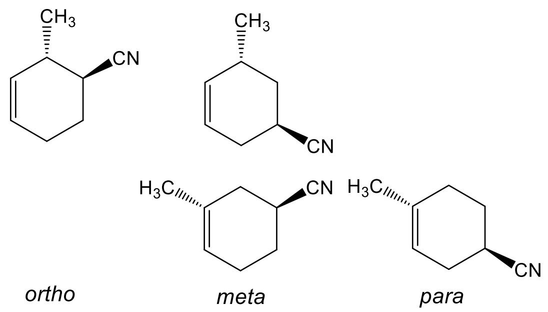

Chart S2. Cycloadducts defining the regioisomeric reaction paths.

2. Study of the P-DA Reaction Between Cp 1 and Acrolein 7

In order to understand the role of the Lewis acid (LA) catalyst in the P-DA reactions of Cp **1** with the series of LA complexes, the P-DA reaction of Cp **1** with acrolein **7** was first analyzed. Due to the non-symmetry of acrolein **7**, two stereoisomeric reaction paths, the *endo* and the *exo*, are feasible. Analysis of the stationary points involved in this P-DA reaction indicates that it takes place along a one-step mechanism. Consequently, the reagents, one molecular complex (MC), **MC**, two stereoisomeric TSs, **TSn** and **TSx**, and the corresponding cycloadducts (CA), **CAn** and **CAx**, were localized and characterized. Relative gas phase electronic energies are given in Scheme S4. Total electronic energies are given in Table S2.

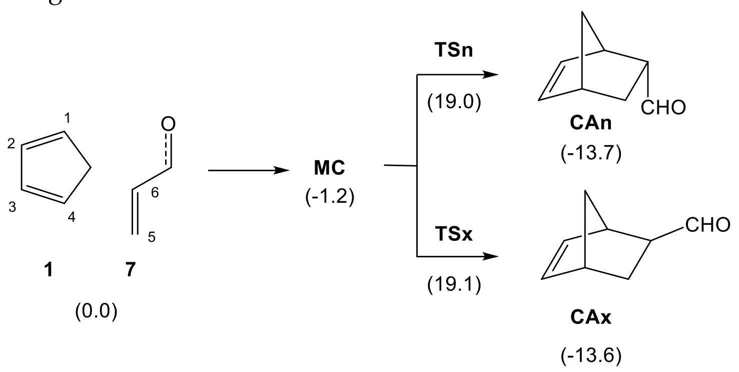

Scheme S4. P-DA reaction of Cp **1** with acrolein **7**. Relative energies, in parentheses, are given in kcal·mol^{-1}.

An exploration of the reaction paths between the separated reagents and the TSs allowed finding an MC in which the two reagents are closer. **MC** is 1.2 kcal·mol^{-1} more stable than separated reagents, being a minimum in their corresponding potential energy surfaces. The activation energies associated to the two stereoisomeric reaction paths are 19.0 (**TSn**) and 19.1 (**TSx**) kcal·mol^{-1}. Consequently, this P-DA reaction is very poor *endo* stereoselective. Formation of the CAs is exothermic by 13.7 (**CAn**) and 13.6 (**CAx**) kcal·mol^{-1}.

The geometries of the two stereoisomeric TSs involved in the P-DA reactions of Cp **1** with acrolein **7** are given in Figure S1. The distance between the C4–C5 and C1–C6 interacting carbons at the two stereoisomeric TSs are: 2.047 and 2.429 Å at **TSn**, and 2.045 and 2.457 Å at **TSn**. These values, which are similar at both TSs, indicate that they are associated to asynchronous C–C bond formation process.

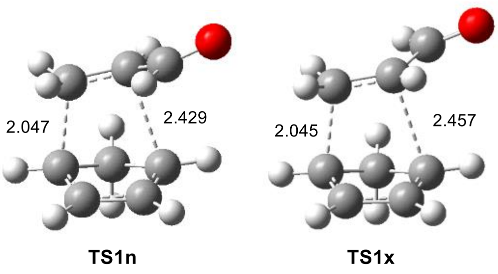

Figure S1. B3LYP/6-311G(d,p) gas phase geometries of the stereoisomeric TSs involved in the P-DA reactions of Cp **1** with acrolein **7**. Distances are given in angstroms, Å.

The polar nature of this DA reaction was evaluated by computing the GEDT [3] at the corresponding TSs. Reactions with GEDT values of 0.0 e correspond to non-polar processes, while values higher than 0.2 e correspond to polar processes. The GEDT values at the corresponding TSs are 0.15 e (**TSn**) and 0.15 e (**TSn**), indicating that this DA reaction has some polar character. Note that the GEDT at the TS associates with the non-polar DA reaction between Cp **1** and ethylene is 0.00 e.

3. BET Study of the P-DA Reaction Between Cp 1 and Complex 7-BF_3

A bonding evolution theory [7] (BET) studying the *endo* reaction path associated with the P-DA reaction between Cp **1** and complex **7-BF_3**, as a reaction model, was performed in order to understand the bonding changes along the reaction path and thus to establish the mechanism of these reactions. A simplified representation of the molecular mechanism by Lewis-like structures arising from the electron localization function [8] (ELF) topology is shown in Scheme S5; the populations of the most significant valence basins, among other relevant parameters, of the selected structures of the intrinsic reaction coordinate (IRC) are gathered in Table S1; the attractor positions of the ELF basins for the structures involved in the bond formation processes are represented in Figure 8 in the manuscript.

The P-DA reaction between Cp **1** and complex **7-BF_3** takes place along ten differentiated phases. The topological features of the ELF of **S1**, which is the first point of the IRC, are very similar to those of the isolated reagents (see Section 2.2. in the manuscript). Along *phase I*, which begins at **S1**, a slight depopulation of the C–C double bond in the reagents is observed. Along *Phase II*, the slight depopulation of the bonding regions of the C–C double bonds present in the reagents is observed. At **S2**, the two V(C1,C2) and V'(C1,C2) disynaptic basins present in Cp **1** have merged into one V(C1,C2) disynaptic basin, integrating 3.23 e. *Phase III* begins at **S3**. The two V(C3,C4) and V'(C3,C4) disynaptic basins present at Cp **1** have merged into one V(C3,C4) disynaptic basin, integrating 3.22 e, as a consequence of the continued depopulation of the C–C double bonds. *Phase IV* starts at **S4**. The two V(C5,C6) and V'(C5,C6) disynaptic basins present at complex **7-BF_3** have merged into one V(C5,C6) disynaptic basin, integrating 3.11 e. The energy cost (EC) associated to the depopulation of the three double bonds along *Phases I–IV* is of 5.5 kcal·mol^{-1}. *Phase V* begins at **S5**. While the population of the V(C1,C2) disynaptic basin has been depopulated by 0.38 e, a new V(C1) monosynaptic basis has been created, with an initial population of 0.28 e. Along *Phases I–V*, while the C1–C2, C3–C4, and C5–C6 double regions are being depopulated, the C2–C3 and C6–C7 ones are being continuously populated. The energy cost (EC) associated to changes along the last phase is of 6.3 kcal·mol^{-1}. *Phase VI* initiates at **S6**. At this point, while the population of the V(C4) monosynaptic basin has been slightly increased to 0.32 e, a new V(C5) monosynaptic basin, with an initial population of 0.19 e, is created. Newly, the electron density of this new monosynaptic basin comes from the depopulation of the C5–C6 bonding region. Note that these monosynaptic basins, which are associated to two C4 and C5 *pseudoradical* centers [9], appear at the most nucleophilic center of Cp **1** and the most electrophilic center of complex **7-BF_3**. In this phase, the TS of the reaction is found. At **TSn-BF_3**, the two V(C4) and V(C5) monosynaptic basins have reached populations of 0.48 and 0.33 e, respectively. *Phase VII* starts at **S7**. The first most relevant topological change along the IRC takes place at **S7**. At this structure, while the V(C4) and V(C5) monosynaptic basins present at **S6** are missing, a new V(C4,C5) disynaptic basin, integrating 0.87 e, is created. This topological change indicates that the formation of the first C4–C5 single bond has begun at a C–C distance of 1.95Å by the C-to-C coupling of the two C4 and C5 *pseudoradical* centers present at **S6** [3]. Along the formation of **S6**, the system releases a molecular relaxation energy (MRE) of ca. 0.12 kcal·mol^{-1}. At **S7**, the maximum of GEDT along the IRC is observed, 0.36 e. *Phase VIII* commences at **S8**. At the beginning of this phase, the V(C4,C5) disynaptic basin has reached a population of 1.58 e. **S8** is characterized by the creation of a new (C1) monosynaptic basin, integrating 0.13 e. On the other hand, the V(C2,C3) disynaptic basin present at **S7** has been split into two new disynaptic basins, V(C2,C3) and V(C2,C3), as a consequence of the

increase of the population in the C2–C3 bonding region by 0.42 e. *Phase IX* begins at **S9**. This phase is characterized by the creation of a new V(C6) monosynaptic basin, integrating 0.33 e. The electron density associated to this *pseudoradical* C6 center comes from the depopulation of the V(C6,C7) disynaptic basin, which has been populated along the reaction path. The last phase, *Phase X* begins at **S10** and end at **CAn-BF$_3$**. At the beginning of this phase, the second most relevant change along the IRC takes place. At this structure, while the V(C1) and V(C6) monosynaptic basins present at **S9** are missing, a new V(C1,C6) disynaptic basin, integrating 0.77 e, is created. These topological changes indicate that the formation of the second C1–C6 single bond has begun at a C–C distance of 2.08 Å by the C-to-C coupling of the two C1 and C6 *pseudoradical* centers present at **S9** [3]. At **CAn-BF$_3$**, the two new V(C4,C5) and (C1,C6) disynaptic basins have reached a population of 1.79 and 1.68 e, respectively. From **S10** to the end of the reaction path, the electron density of the **S10** is relaxed, releasing an MRE of ca. 15.1 kcal·mol^{-1}.

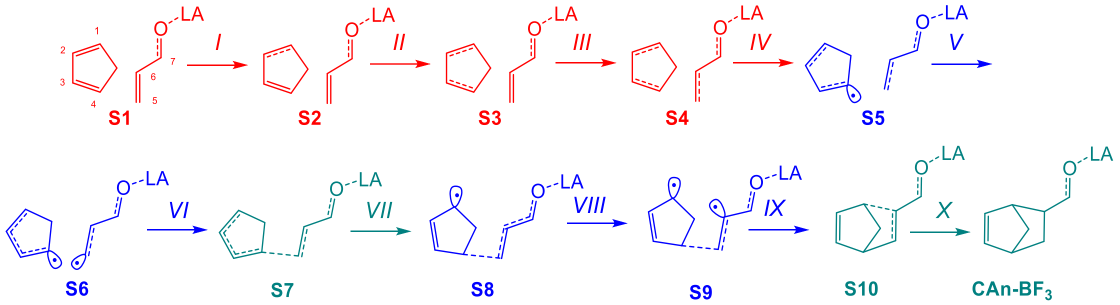

Scheme S5. Simplified representation of the molecular mechanism of the P-DA reaction between Cp 1 and 7-BF3 complex by Lewis-like structures arising from the topological analysis of the electron localization function (ELF) along the reaction path. In red are marked the phases associated with the rupture of the C–C double bonds, in blue are marked the phases associated with the formation of the pseudoradical carbons, and in green are marked the phases associated with the formation of the new C–C single bonds. Atom numbering is included.

Table S1. ELF valence basin populations, distances of the forming bonds, B3LYP/6-311G(d) relative[a] gas phase electronic energies, GEDT of the IRC structures, **S1**–**S10**, defining the ten phases characterizing the molecular mechanism of the P-DA reactions between Cp **1** and complex **7-BF$_3$**. **TSn-BF$_3$,** and **CAn-BF$_3$** are also included. Distances are given in angstroms, Å, GEDT values, and electron populations in average number of electrons, e, relative energies in kcal·mol^{-1}.

**Structures**	**S1**	**S2**	**S3**	**S4**	**S5**	**S6**	**TSn-BF$_3$**	**S7**	**S8**	**S9**	**S10**	**CAn-BF$_3$**
Phases	I	II	III	IV	V		VI	VII	VIII	IX	X	
d(C4-C5)	3.501	3.111	2.940	2.544	2.120	2.084	1.977	1.954	1.636	1.625	1.609	1.569
d(C1-C6)	3.485	3.277	3.180	2.954	2.774	2.761	2.722	2.713	2.310	2.229	2.088	1.600
GEDT	0.05	0.09	0.11	0.18	0.30	0.32	0.35	0.36	0.35	0.32	0.27	0.08
ΔE	0.0	0.7	1.5	5.5	11.8	12.1	12.6	12.6	7.6	6.6	4.3	−10.8
V(C1,C2)	1.55	3.23	3.19	3.13	2.96	2.93	2.86	2.84	2.45	2.39	2.28	2.01
V'(C1,C2)	1.72											
V(C2,C3)	2.25	2.27	2.30	2.36	2.55	2.57	2.67	2.69	1.66	1.54	1.58	1.76
V'(C2,C3)									1.45	1.63	1.67	1.68
V(C3,C4)	1.61	1.56	3.22	3.13	2.75	2.70	2.54	2.50	2.13	2.11	2.08	2.00
V'(C3,C4)	1.70	1.71										
V(C5,C6)	1.60	1.59	1.57	3.11	3.00	2.80	2.62	2.57	2.13	2.11	2.06	1.84
V'(C5,C6)	1.59	1.58	1.59									
V(C4)					0.28	0.32	0.48					
V(C5)						0.19	0.33					
V(C4,C5)								0.87	1.58	1.62	1.67	1.79
V(C1)									0.13	0.18		
V(C6)										0.33		
V(C1,C6)											0.77	1.68
V(C6,C7)	2.35	2.38	2.40	2.47	2.67	2.69	2.79	2.82	3.01	2.68	2.54	2.19

[a] Relative to the first structure of the IRC, **S1.**

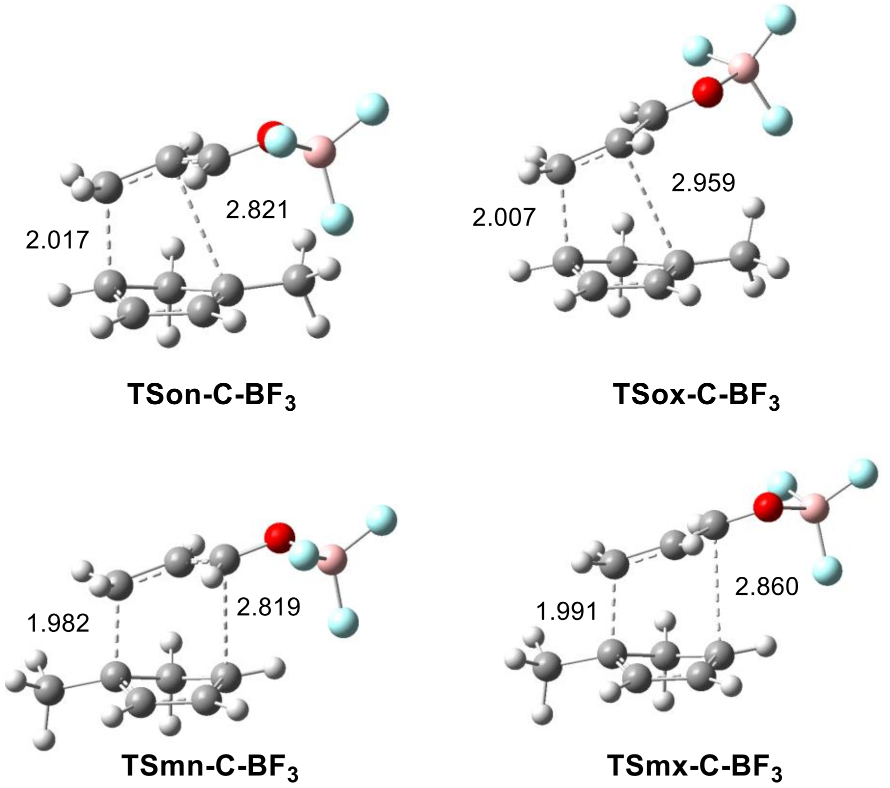

Figure S2. B3LYP/6-311G(d,p) gas phase geometries of the regio- and stereoisomeric transition state structures (TSs) and involved in t$_3$he P-DA reaction of Cp **12** with complex **7-BF$_3$**. Distances are given in angstroms, Å.

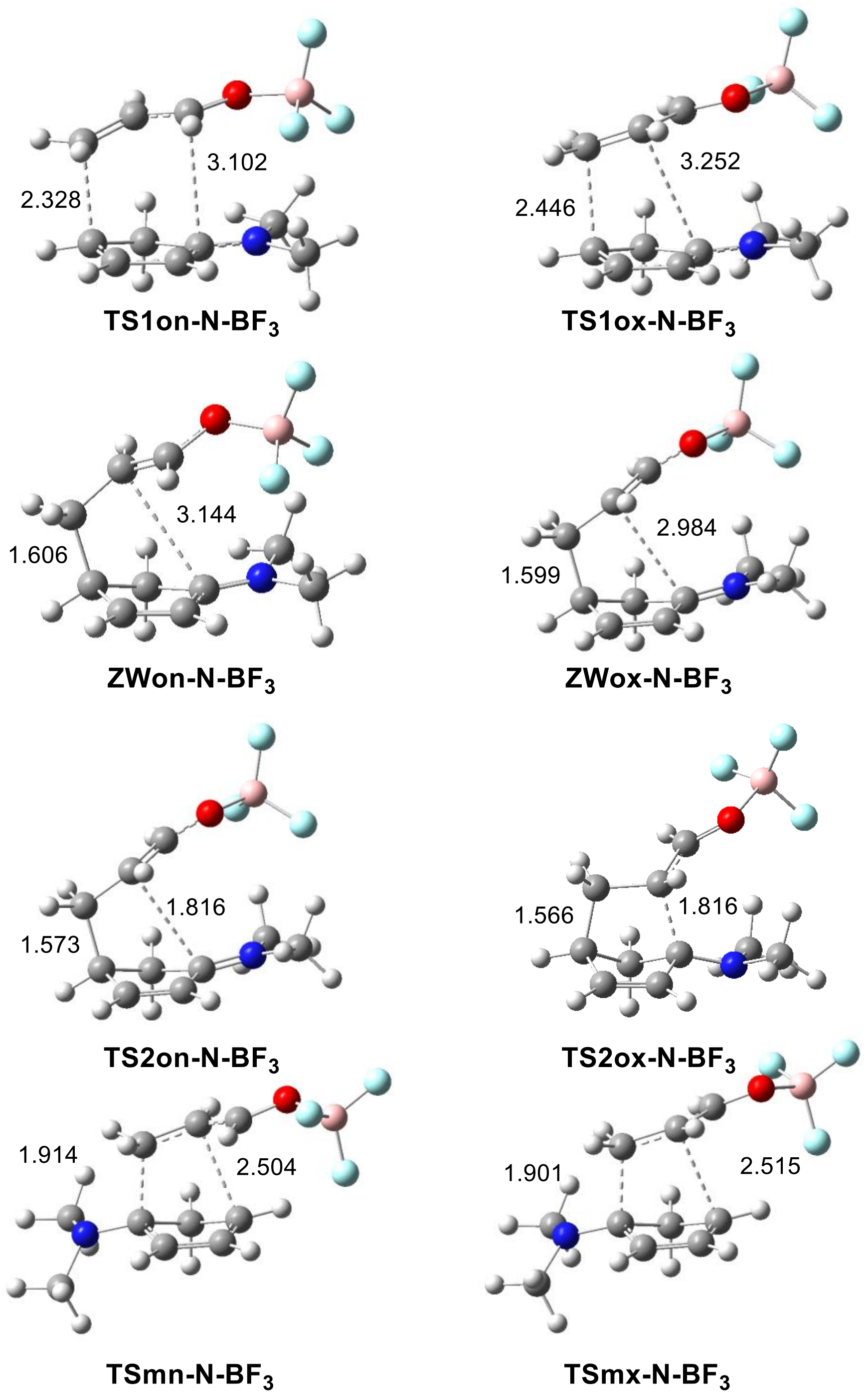

Figure S3. B3LYP/6-311G(d,p) gas phase geometries of the regio- and stereoisomeric TSs and intermediates involved in the P-DA reaction of Cp **14** with complex **7-BF**$_3$. Distances are given in angstroms, Å.

Table S2. B3LYP/6-311G(d,p) gas phase total energies, in a.u., of the stationary points involved in the P-DA reactions between Cp **1** and acrolein **7**, and the series of Lewis acid (LA) complexes.

LA complex	7	7-BH3	7-BF3
**Cp**	−194.1537237		
**7-LA**	−191.9682515	−218.6191346	−516.639503
**MC-LA**	−386.123899	−412.7760850	−710.799137
**TSn-LA**	−386.0917272	−412.7521315	−710.778414
**TSx-LA**	−386.0916133	−412.7518093	−710.778107
**CAn-LA**	−386.1438248	−412.7952882	−710.816188
**CAx-LA**	−386.1437012	−412.7948052	−710.816637
**LA complex**	**7-AlMe3**	**7-TiCl4**	**7-AlCl3**
**CHO**	−554.248132	−2882.618880	−1815.356694
**MC-LA**	−748.404953	−3076.776497	−2009.517092
**TSn-LA**	−748.382382	−3076.756895	−2009.502158
**TSx-LA**	−748.382080	−3076.756427	−2,009.501480
**CAn-LA**	−748.424506	−3076.795762	−2009.534054
**CAx-LA**	−748.424058	−3076.795294	−2009.533564

Table S3. B3LYP/6-311G(d,p) gas phase total energies, in a.u., of the stationary points involved in the P-DA reactions of Cps **12–14** with complex **7-BF$_3$**.

X	12<br>C	13<br>O	14<br>N
**Cp**	−233.483418	308.709950	328.158843
**7-BF$_3$**	−516.639503		
**CM-X-BF$_3$**	−750.130127	−825.359015	−844.814769
**TS1on-X-BF$_3$**	−750.115040	−825.350985	−844.813267
**TS1ox-X-BF$_3$**	−750.113645	−825.346786	−844.812208
**ZWon-X-BF$_3$**		−825.359966	−844.831643
**ZWox-X-BF$_3$**		−825.356889	−844.835006
**TS2on-X-BF$_3$**		−825.328615	−844.776024
**TS2ox-X-BF$_3$**		−825.328318	−844.776168
**TSmn-X-BF$_3$**	−750.105982	−825.357924	−844.812935
**TSmx-X-BF$_3$**	−750.105810	−825.356342	−844.812285
**CAon-X-BF$_3$**	−750.144638	−825.365472	−844.810375
**CAox-X-BF$_3$**	−750.143507	−825.365937	−844.813157
**CAmn-X-BF$_3$**	−750.143771	−825.365178	−844.810438
**CAmx-X-BF$_3$**	−750.144130	−825.365048	−844.810180

Table S4. B3LYP/6-311G(d,p) total energies, E in a.u., enthalpies , H in a.u., entropies, ΔS in cal·mol^{-1}K^{-1}, and Gibbs-free energies, G in a.u., computed at 25 °C in DCM, of the stationary points involved in the P-DA reaction of Cp **13** with complex **7-BF$_3$**.

	E	H	S	G
**13**	−308.713294	−308.581821	77.4	−308.615521
**7-BF$_3$**	−516.654324	−516.570070	88.1	−516.608410
**MC-O-BF$_3$**	−825.373497	−825.155726	128.0	−825.211422
**TS1on-O-BF$_3$**	−825.371316	−825.153757	119.7	−825.205883
**TS1ox-O-BF$_3$**	−825.368229	−825.150843	120.5	−825.203299
**ZWon-O-BF$_3$**	−825.387848	−825.167561	119.3	−825.219490
**ZWox-O-BF$_3$**	−825.385050	−825.164855	123.9	−825.218775
**TS2on-O-BF$_3$**	−825.376044	−825.156582	113.3	−825.205889
**TS2ox-O-BF$_3$**	−825.375972	−825.156612	114.3	−825.206359
**TSmn-O-BF$_3$**	−825.350979	−825.133475	120.2	−825.185787
**TSmx-O-BF$_3$**	−825.351134	−825.133757	119.8	−825.185904
**CAon-O-BF$_3$**	−825.379901	−825.159306	117.8	−825.210601
**CAox-O-BF$_3$**	−825.380481	−825.159868	117.5	−825.211029
**CAmn-O-BF$_3$**	−825.379491	−825.158863	117.7	−825.210081
**CAmx-O-BF$_3$**	−825.379539	−825.158891	117.5	−825.210044

4. References

1. Domingo, L.R.; Sáez, J.A. Understanding the Mechanism of the Polar Diels-Alder Reaction *Org. Biomol. Chem.* **2009**, *7*, 3576–3583.
2. Domingo, L.R.; Ríos-Gutiérrez, M.; Pérez, P. A molecular electron density theory study of the participation of tetrazines in aza-Diels–Alder reactions. *RSC Adv.* **2020**, *10*, 15394–15405
3. Domingo, L.R. A new C-C bond formation model based on the quantum chemical topology of electron density. *RSC Adv.* **2014**, *4*, 32415–32428.
4. Domingo, L.R.; Ríos-Gutiérrez, M.; Pérez, P. Applications of the conceptual density functional indices to organic chemistry reactivity. *Molecules* **2016**, *21*, 748.
5. Domingo, L.R.; Sáez, J.A.; Zaragozá, R.J.; Arnó, M. Understanding the Participation of Quadricyclane as Nucleophile in Polar [2σ + 2σ + 2π] Cycloadditions toward Electrophilic π Molecules. *J. Org. Chem.* **2008**, *73*, 8791–8799.
6. Domingo, L.R.; Ríos-Gutiérrez, M.; Pérez. P. A Molecular Electron Density Theory Study of the Competitiveness of Polar Diels-Alder and Polar Alder Ene Reactions. *Molecules* **2018**, *23*, 1913.
7. Krokidis, X.; Noury, S.; Silvi, B. Characterization of Elementary Chemical Processes by Catastrophe Theory *J. Phys. Chem. A* **1997**, *101*, 7277–7282.
8. Becke, A.D.; Edgecombe, K.E. A simple measure of electron localization in atomic and molecular-systems. *J. Chem. Phys.* **1990**, *92*, 5397–5403.
9. Domingo, L. R.; Chamorro, E.; Pérez, P. Understanding the High Reactivity of the Azomethine Ylides in [3 + 2] Cycloaddition Reactions. *Lett. Org. Chem.* **2010**, *7*, 432-439.
